# COVID-19 vaccine-induced Recurrence of the Radiation Recall Phenomenon in the Laryngeal Mucosa Due to a VEGF Inhibitor

**DOI:** 10.1016/j.adro.2022.101048

**Published:** 2022-08-14

**Authors:** Shotaro Tatekawa, Shigenori Hoshino, Norihiko Takemoto, Michio Oda, Yuichi Akino, Kota Iwahori, Takero Hirata, Kazuhiko Hayashi, Keisuke Tamari, Yuji Seo, Fumiaki Isohashi, Shinichi Shimizu, Kazuhiko Ogawa

**Affiliations:** a Department of Radiation Oncology, Osaka University Graduate School of Medicine, Osaka, Japan; b Department of Respiratory Medicine, Saito Yukoukai Hospital, Osaka, Japan; c Department of Otorhinolaryngology, Saito Yukoukai Hospital, Osaka, Japan; d Department of Otorhinolaryngology-Head and Neck Surgery, Osaka University Graduate School of Medicine, Osaka, Japan; e Department of Medical Technology, Osaka University Hospital, Osaka, Japan; f Department of Respiratory Medicine and Clinical Immunology, Osaka University Graduate School of Medicine, Osaka, Japan

## Abstract

**Purpose:**

The radiation recall phenomenon (RRP) is a rare and unexpected late complication of radiation therapy (RT). Although predominantly in the skin, RRP of the upper respiratory tract has also been reported. In general, RRP is caused by anticancer agents, and the COVID-19 vaccine has also been reported to cause RRP in recent years.

**Methods and Materials:**

A 50-year-old woman who had received RT around the larynx 3 years prior and was receiving a docetaxel + ramucirumab (RAM) regimen experienced recurrent sore throat. The administration of RAM was discontinued after a gastroscopic examination revealed mucosal bleeding from around the larynx, which was thought to be RRP caused by RAM, a vascular endothelial growth factor inhibitor.

**Results:**

After the remission of the RRP, the patient received a COVID-19 vaccine (Pfizer-BioNTech). Five days later, the appearance of cough and recurrence of sore throat worsened with time, and marked stridor was observed. The patient was admitted, and steroid pulse therapy was administered for 3 days starting on day 18 after vaccination. On day 50 after vaccination, edema of the vocal cords improved.

**Conclusions:**

When administering COVID-19 vaccines, considering that these vaccines may cause RRP is important, because RRP can be fatal in patients with a history of RT in the laryngeal region and treated with vascular endothelial growth factor inhibitors.

## Introduction

The radiation recall phenomenon (RRP) can unexpectedly occur in patients who receive systemic therapy after a course of radiation therapy (RT). The late effect is mainly reported as an acute skin reaction, which usually appears >1 week after completion of RT. In addition to the skin, onset of inflammation in the lungs and central nervous system has been reported.^1^ The RRP is induced by anticancer antibiotics, alkylating agents, antimetabolites, microtubule inhibitors, and molecular target drugs. The disease's cause is unclear, but some immune system involvement has been suggested. In recent years, RRP of the skin caused by COVID-19 vaccination was first reported by Soyfer et al.^2^ The RRP's mechanism is not clear, but also thought to be related to the response to inflammation.

Although there are only a few reports of the RRP in the upper airway, RRP in the laryngeal region has been reported for a long time,^3^ In recent years, RRP in the upper aerodigestive tract has been reported to recur due to stimulation by anticancer drugs, including vascular endothelial growth factor (VEGF) inhibitors.^4^ To the best of our knowledge, this is the first report of a COVID-19 vaccine-induced recurrence of the RRP in the laryngeal mucosa caused by a VEGF inhibitor.

## Case presentation

**Figure 1:**
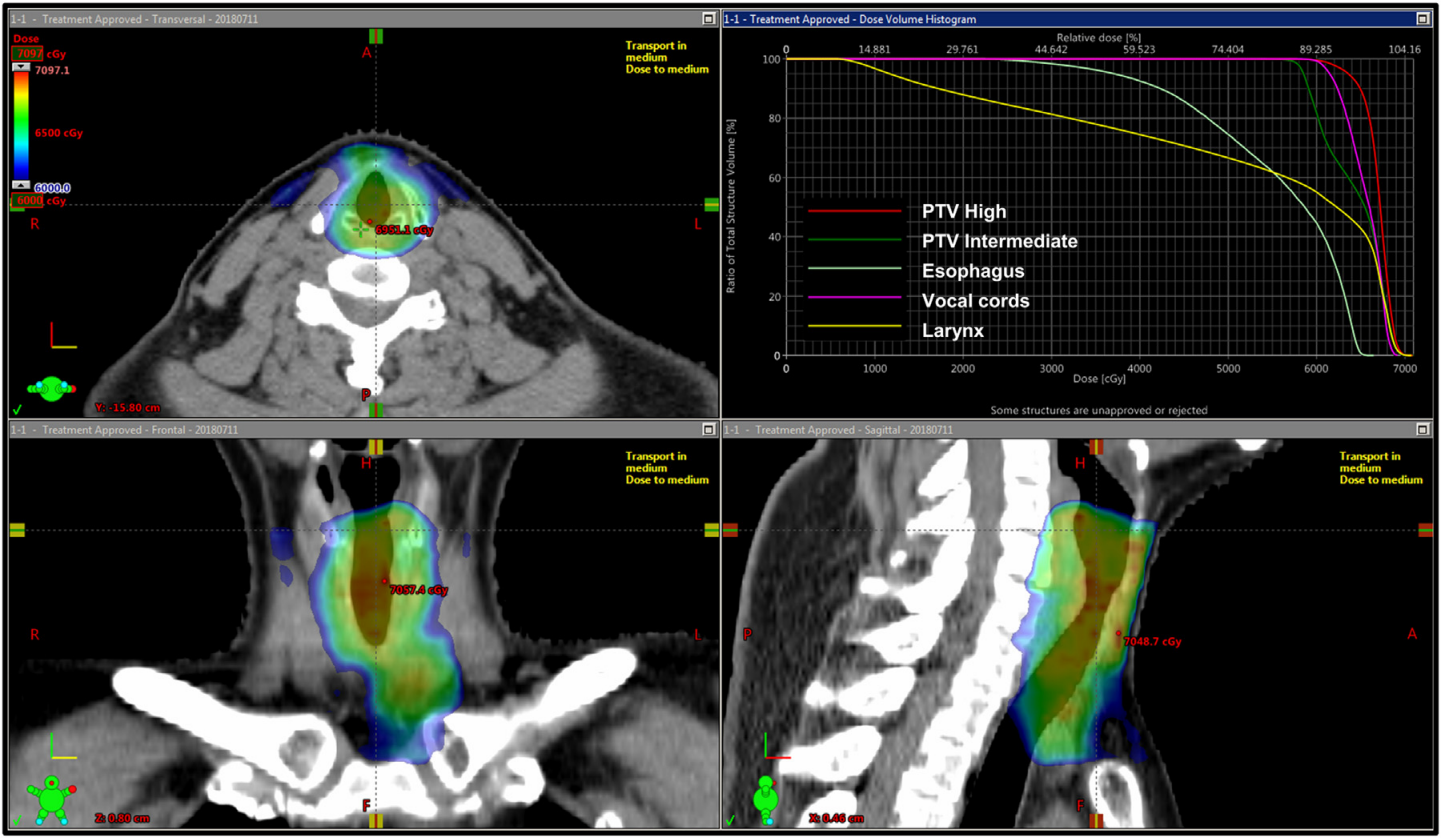
Radiation treatment plan for oligometastasis of postoperative lung adenocarcinoma.

**Figure 2:**
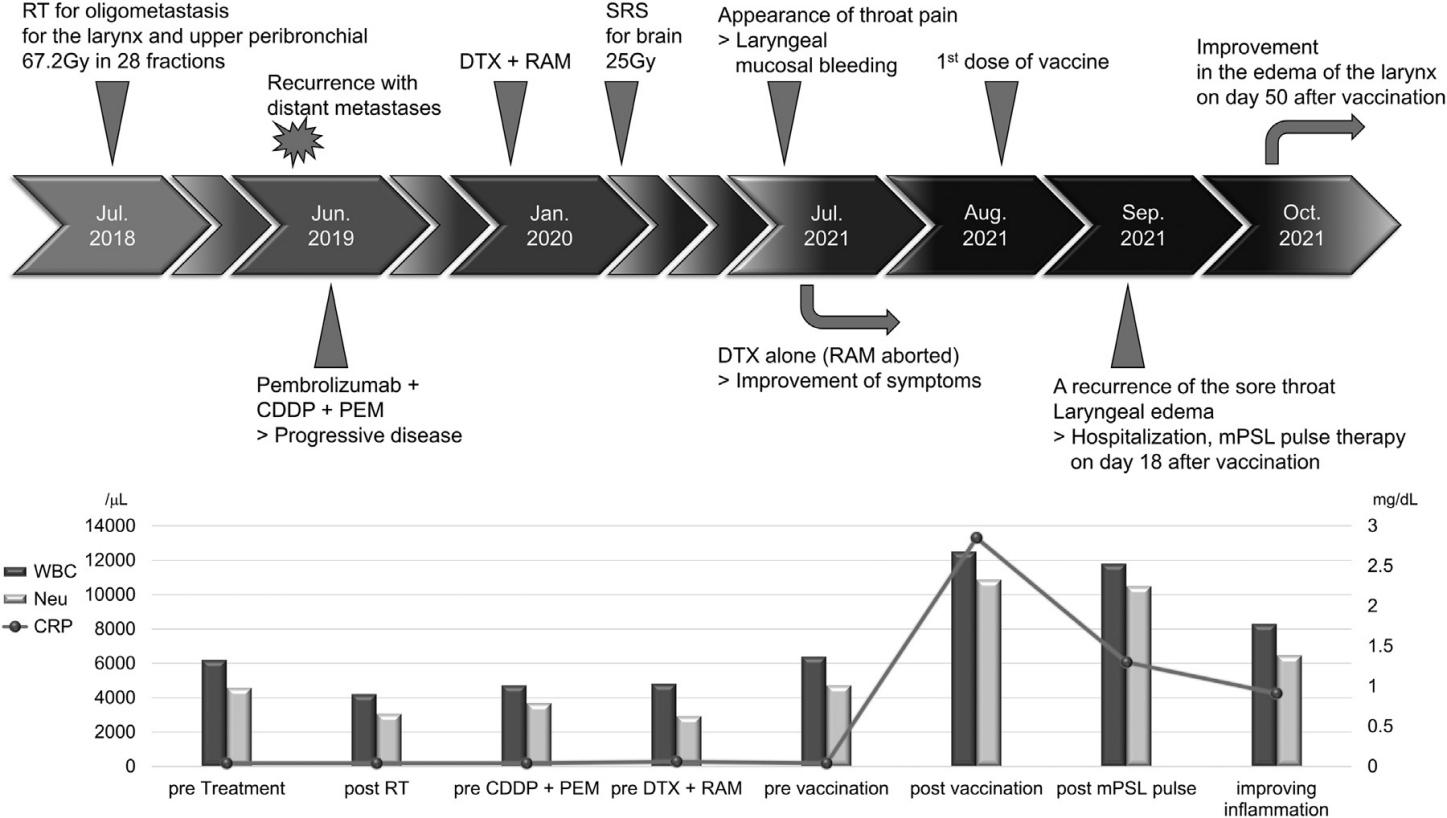
Timeline for case.

A 50-year-old woman received RT at a dose of 67.2 Gy in 28 fractions to the larynx and upper peribronchial region for postoperative recurrence (oligometastasis) of lung adenocarcinoma 3 years prior (Fig. 1). Complications of laryngeal mucositis grade 2 and radiation dermatitis grade 1 were observed, but spontaneously resolved after irradiation. Detailed irradiation information is shown in Supplementary Figure 1, and Figure 2 shows the time course for the patient. Unfortunately, the patient relapsed with distant metastases about 1 year later, and began treatment with a regimen of docetaxel (DTX) + ramucirumab (RAM) 1 year and 6 months after RT. Simultaneously, stereotactic radiosurgery at a dose of 25 Gy was performed for brain metastasis (Fig. E2).

Although no late complications, such as laryngeal edema, occurred (Figs. E3 A and B), 1 year and 4 months after the start of DTX + RAM, the patient had a recurrence of sore throat similar to the laryngeal mucositis experienced during RT. A gastroscopic examination revealed mucosal bleeding from around the larynx, which was thought to be the RRP caused by RAM, the administration of which was subsequently aborted (Fig. E3C). After switching to DTX alone, the patient's sore throat did not flare up again, and the RRP was in remission. After 2 courses of DTX alone, the patient received a COVID-19 vaccine (Pfizer-BioNTech). Five days later, the appearance of cough and recurrence of sore throat worsened with time, and marked stridor was observed. Therefore, the patient was referred to the department of otolaryngology.

**Figure 3:**
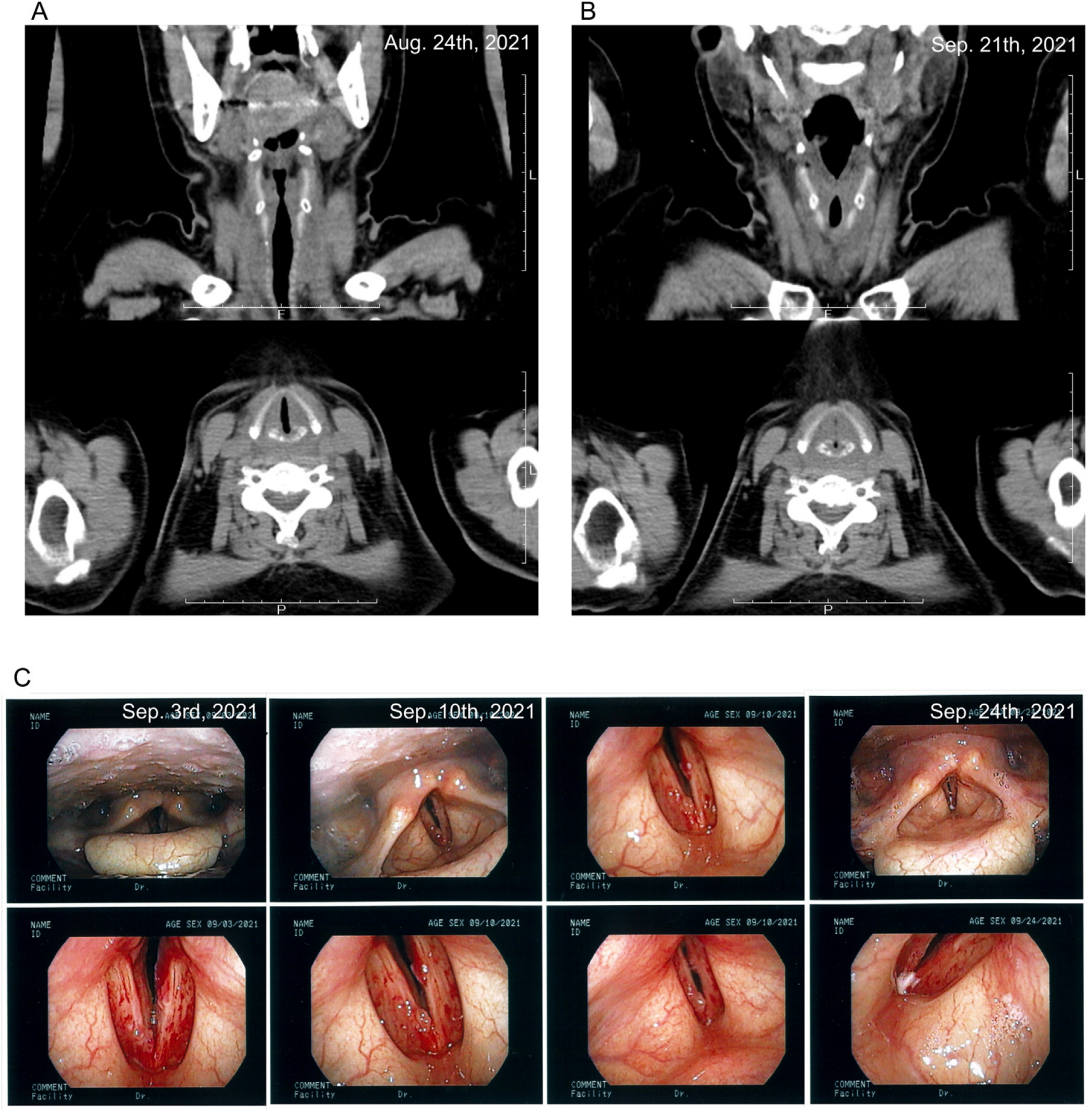
Computed tomography imaging and nasopharyngoscopy findings after COVID-2019 vaccination, showing computed tomography images at A, 5 days after COVID-19 vaccination and B, 2 weeks after steroid pulse therapy, as well as C, nasopharyngoscopy images before and after steroid pulse therapy.

**Figure 4:**
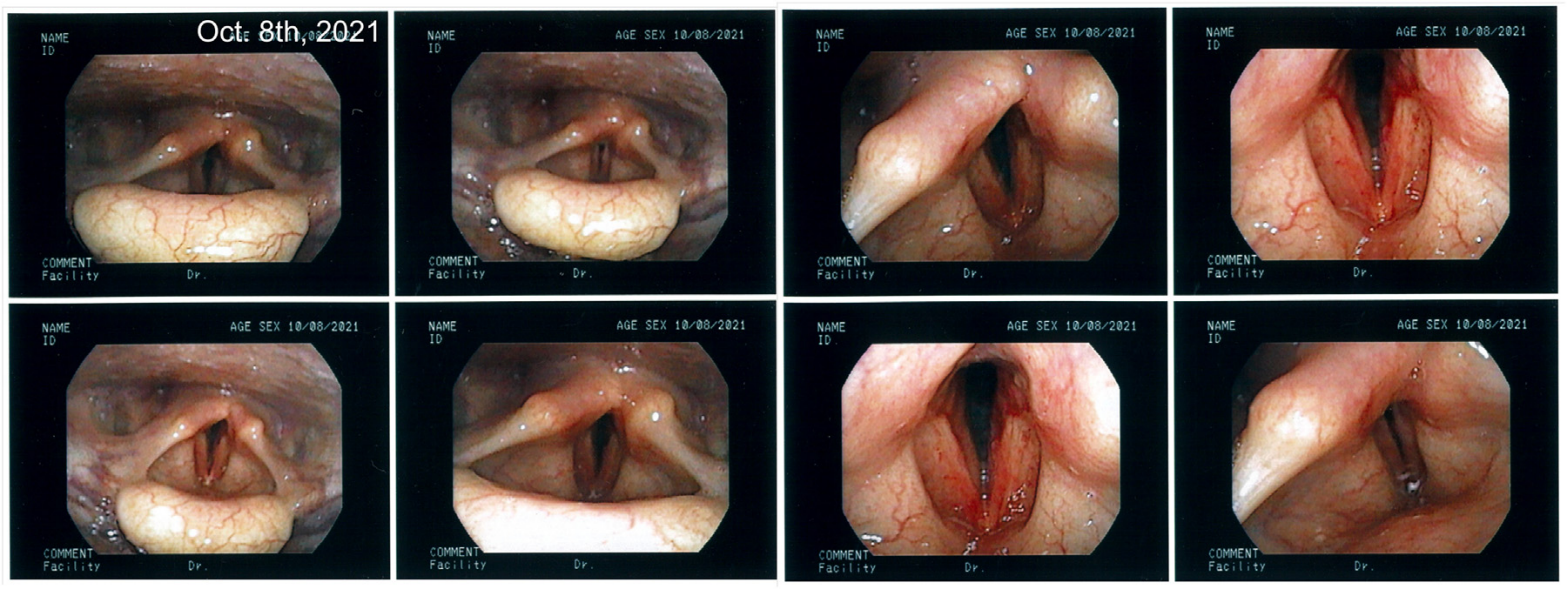
Nasopharyngoscopy image showing relief of edema.

Computed tomography (CT) and nasopharyngoscopy revealed swollen vocal cords and edema of the surrounding mucosa over the subglottis (Figs. 3A and C [left]). Since there was a risk of needing a tracheotomy if the edema worsened, the patient was managed at the hospital, and steroid pulse therapy (methylprednisolone 500 mg by intravenous injection) was administered for 3 days starting on day 18 after vaccination. Two weeks after steroid pulse therapy, the CT and nasopharyngoscopy findings (Figs. 3B and C [right]) did not show considerable improvement in the edema of the vocal cords (rather slightly worsened), but after another 2 weeks (day 50 after vaccination), edema of the vocal cords improved (Fig. 4). A time course of the blood examination is shown in the lower part of Figure 2. There was no increase in inflammatory response before and after RT and chemotherapy, including DTX+RAM, but after the first vaccination, there was an increase in inflammatory response (C-reactive protein: 0.04-2.85 mg/dL) and white blood cell/neutrophil count (6400/4740-12500/10880/μL). After steroid pulse therapy, both parameters showed a decreasing trend with improvement in the inflammatory findings in the larynx. Up until the last follow up, the area around the larynx where RT was administered was recurrence-free. The patient involved in this case report provided informed consent.

## Discussion

The COVID-19 vaccines were developed to overcome the SARS-CoV-2 viral pandemic, and have had some effect on infection control. However, side effects of the vaccine, such as myocarditis, have been reported,^5^ and cases of vaccine-induced RRP have been reported as well one after another (Table 1). As in the case of anticancer drug-induced RRP, skin reactions are the most frequently reported symptoms,^2^^,^^6^^,^^7^ but there are also reports of pneumonia.^8^, ^9^, ^10^ There are some reports of patients being treated with molecularly targeted agents or immune checkpoint inhibitors, which may have modified a vaccination-induced inflammatory or immune response. The present case differs from previous RRP cases in 3 unique aspects: the risk of fatality was caused by edema of the laryngeal mucosa, the vaccine was used in combination with a VEGF inhibitor, and the RRP (induced because of RAM and subsided after RAM discontinuation) flared up after vaccination.

**Table 1:** Review of reported cases with RRP by COVID-2019 vaccine

Author, y	Region of RRP	Age/sex	RT plan	Type of vaccine	Period between RT and RRP	Dose of vaccination	Period between vaccination and RRP	Treatment prior to vaccination	Reference
Soyfer et al, 2021	Skin (2 cases)	68/M; 64/M	50 Gy/25 fx; 39 Gy/13 fx	Pfizer-BioNTech (both)	8 mo;1.5 mo	2^nd^; 2^nd^	5 d; 6 d	None	^2^
Stewart et al, 2021	Skin	57/F	66 Gy/33 fx	AstraZeneca	6 mo	1^st^	3 h-3 wk (worsening)	None	^6^
Afacan et al, 2021	Skin	60/F	30 Gy/10 fx	Sinovac	2 y 3 mo	1^st^	5 d	Dabrafenib + trametinib	^7^
Hughes et al, 2021	Lung	67/M	60 Gy/15 fx	Not available (mRNA vaccine)	1.5 y	2^nd^	4 d	None	^8^
Shinada et al, 2021	Lung	48/M	60 Gy/30 fx	Pfizer-BioNTech	1 y	2^nd^	19 d	Durvalumab	^9^
Steber et al, 2021	Lung	66/M	45 Gy/15 fx	Moderna	8 mo	2^nd^	5 d	Pemetrexed + pembrolizumab	^10^
Present case	Larynx	50/F	67.2 Gy/28 fx	Pfizer-BioNTech	3 y	1^st^	5 d-2 wk (worsening)	Docetaxel + ramucirumab	Present case

F, female; fx, fraction; M, male; mRNA, messenger ribonucleic acid; RRP, radiation recall phenomenon; RT, radiation therapy

The side effects associated with the combined use of VEGF inhibitors and RT have been previously discussed.^11^^,^^12^ Although the combination of RT with bevacizumab for pancreatic cancer was reported as feasible,^13^ clinical trials regarding side effects on the airway mucosa have been discontinued due to tracheoesophageal fistulas in patients with lung cancer treated with bevacizumab and chemoradiation.^14^ Similar events have been reported with RAM.^15^ There may be a difference in the sensitivity of the gastrointestinal tract and airway mucosa to the combination of VEGF inhibitors and radiation. With the recent advent of molecular target-based agents, there have been reports of RRP caused by bevacizumab, as in the present case.^16^ Gastrointestinal bleeding has also been reported in patients treated with bevacizumab after RT,^17^ which may be related to the RRP.

The RRP's exact pathogenesis remains unclear. Some investigators have suggested that RRP may be the result of vascular damage, epithelial stem cell sensitivity, or drug hypersensitivity of the irradiation field, in addition to upregulated inflammation-mediating cytokines induced by chemotherapeutic agents.^1^^,^^3^ The pathologic evaluation of the RRP on the skin caused by the COVID-19 vaccine showed epidermal intercellular edema, lymphocyte exocytosis, rare necrotic keratinocytes, and increased dermal collagenization and fibrosis, suggesting the intervention of immunocompetent cells to inflammation.^7^ Inflammatory cytokines and vascular endothelial damage-related cytokines, including VEGF, are elevated in SARS-CoV-2 infection, and have been reported to be associated with severe disease.^18^ Whether the COVID-19 vaccination caused an increase in cytokines, such as VEGF, is unclear, but at least its side effect myocarditis is thought to be partly due to the involvement of elevated inflammatory cytokines.^5^ In the present case, the RRP due to a VEGF inhibitor relapsed by the vaccine with an increased inflammatory response, suggesting the involvement of cytokines elicited by the vaccine-induced immune response.

Of note, the RRP occurs primarily on the skin, but can also occur in the mucosa of the upper respiratory tract, especially with the use of VEGF inhibitors, and can also be induced by the COVID-19 vaccine. Clinical trials using VEGF inhibitors for recurrent head and neck cancers, including patients with prior RT, have also been recently conducted.^19^ Furthermore, although the COVID-19 vaccination booster is expected to be promoted worldwide, the RRP can be fatal in patients with a history of RT in the laryngeal region and treated with VEGF inhibitors.

## Appendix Supplementary materials

Image, image 1

Image, image 2

Image, image 3

## References

[1] Burris HA, Hurtig J. (2010). Radiation recall with anticancer agents. Oncologist.

[2] Soyfer V, Gutfeld O, Shamai S, Schlocker A, Merimsky O. (2021). COVID-19 vaccine-induced radiation recall phenomenon. Int J Radiat Oncol Biol Phys.

[3] Azria D, Magné N, Zouhair A (2005). Radiation recall: A well recognized but neglected phenomenon. Cancer Treat Rev.

[4] McGrath LA, Martenson JA, Finley RR. (2019). Recurrent radiation recall mucosal toxicity of the upper aerodigestive tract: A case report. Adv Radiat Oncol.

[5] Bozkurt B, Kamat I, Hotez PJ. (2021). Myocarditis with COVID-19 mRNA vaccines. Circulation.

[6] Stewart R, McDowell L. (2021). Radiation recall phenomenon following COVID-19 vaccination. Int J Radiat Oncol Biol Phys.

[7] Afacan E, Öğüt B, Üstün P, Şentürk E, Yazıcı O, Adışen E. (2021). Radiation recall dermatitis triggered by inactivated COVID-19 vaccine. Clin Exp Dermatol.

[8] Hughes NM, Hammer MM, Awad MM, Jacene HA. (2022). Radiation recall pneumonitis on FDG PET/CT triggered by COVID-19 vaccination. Clin Nucl Med.

[9] Shinada K, Murakami S, Yoshida D, Saito H. (2022). Radiation recall pneumonitis after COVID-19 vaccination. Thorac Cancer.

[10] Steber CR, Ponnatapura J, Hughes RT, Farris MK. (2021). Rapid development of clinically symptomatic radiation recall pneumonitis immediately following COVID-19 vaccination. Cureus.

[11] Niyazi M, Maihoefer C, Krause M, Rödel C, Budach W, Belka C. (2011). Radiotherapy and “new” drugs-new side effects?. Radiat Oncol.

[12] Pollom EL, Deng L, Pai RK (2015). Gastrointestinal toxicities with combined antiangiogenic and stereotactic body radiation therapy. Int J Radiat Oncol Biol Phys.

[13] Small W, Mulcahy MF, Rademaker A (2011). Phase II trial of full-dose gemcitabine and bevacizumab in combination with attenuated three-dimensional conformal radiotherapy in patients with localized pancreatic cancer. Int J Radiat Oncol Biol Phys.

[14] Spigel DR, Hainsworth JD, Yardley DA (2010). Tracheoesophageal fistula formation in patients with lung cancer treated with chemoradiation and bevacizumab. J Clin Oncol.

[15] Lee YL, Hsu JF, Yang CJ. (2019). Tracheoesophageal fistula in a patient with advanced non-small cell lung cancer who received chemoradiotherapy and ramucirumab. J Thorac Oncol.

[16] Saif MW, Ramos J, Knisely J. (2008). Radiation recall phenomenon secondary to bevacizumab in a patient with pancreatic cancer. JOP.

[17] Lordick F, Geinitz H, Theisen J, Sendler A, Sarbia M. (2006). Increased risk of ischemic bowel complications during treatment with bevacizumab after pelvic irradiation: Report of three cases. Int J Radiat Oncol Biol Phys.

[18] Rovas A, Osiaevi I, Buscher K (2021). Microvascular dysfunction in COVID-19: The mystic study. Angiogenesis.

[19] Argiris A, Li S, Savvides P (2019). Phase III randomized trial of chemotherapy with or without bevacizumab in patients with recurrent or metastatic head and neck cancer. J Clin Oncol.

