# Supplementary figures and images for: COVID-19 vaccine-induced Recurrence of the Radiation Recall Phenomenon in the Laryngeal Mucosa Due to a VEGF Inhibitor
Source: Adv Radiat Oncol. 2022 Aug 14;7(6):101048. doi: 10.1016/j.adro.2022.101048 (PMC9376028; doi:10.1016/j.adro.2022.101048)

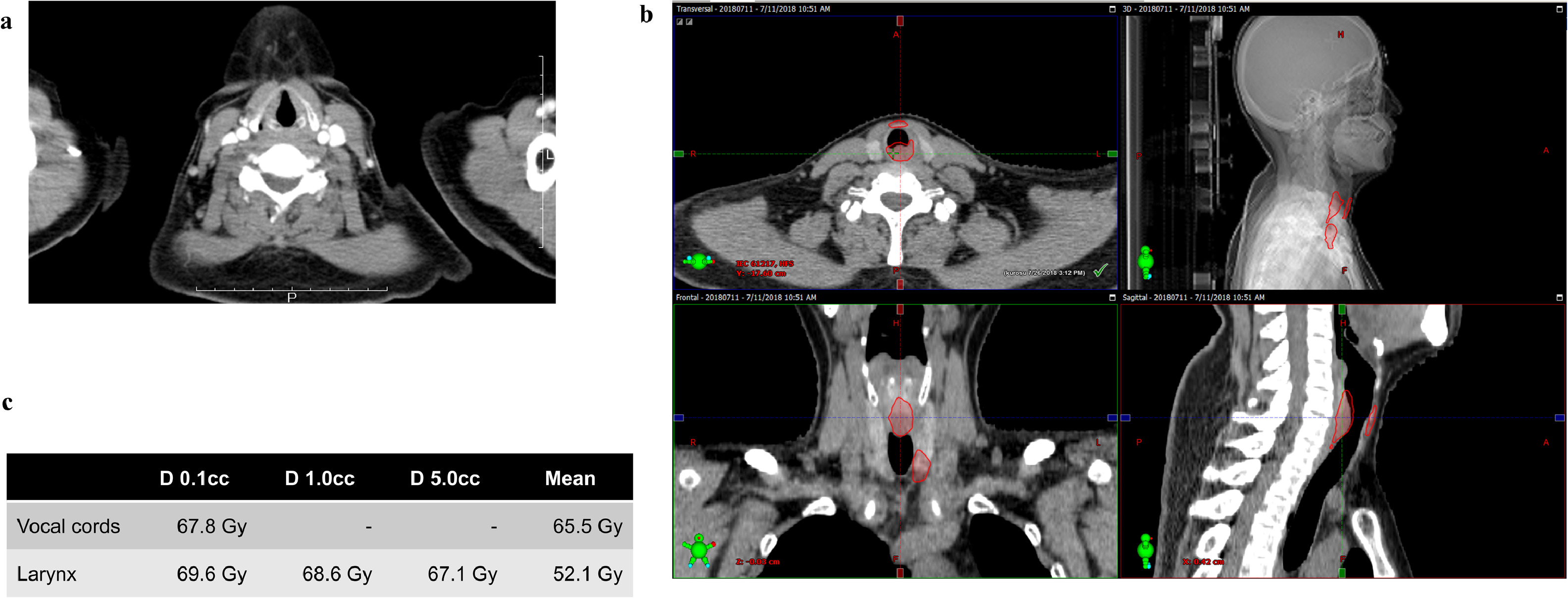

Supplement: Supplementary file 1 [file mmc1.jpg]

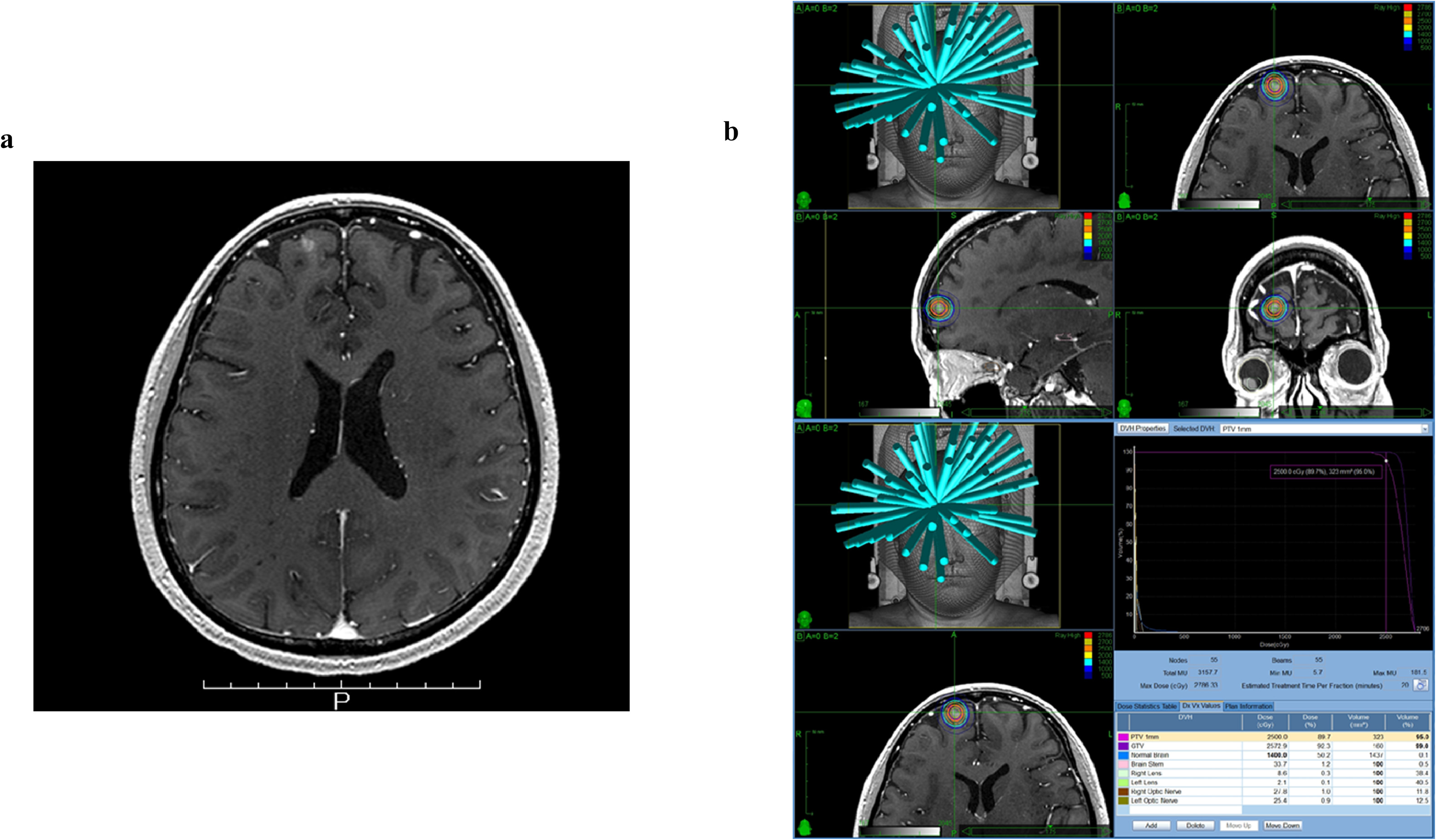

Supplement: Supplementary file 2 [file mmc2.jpg]

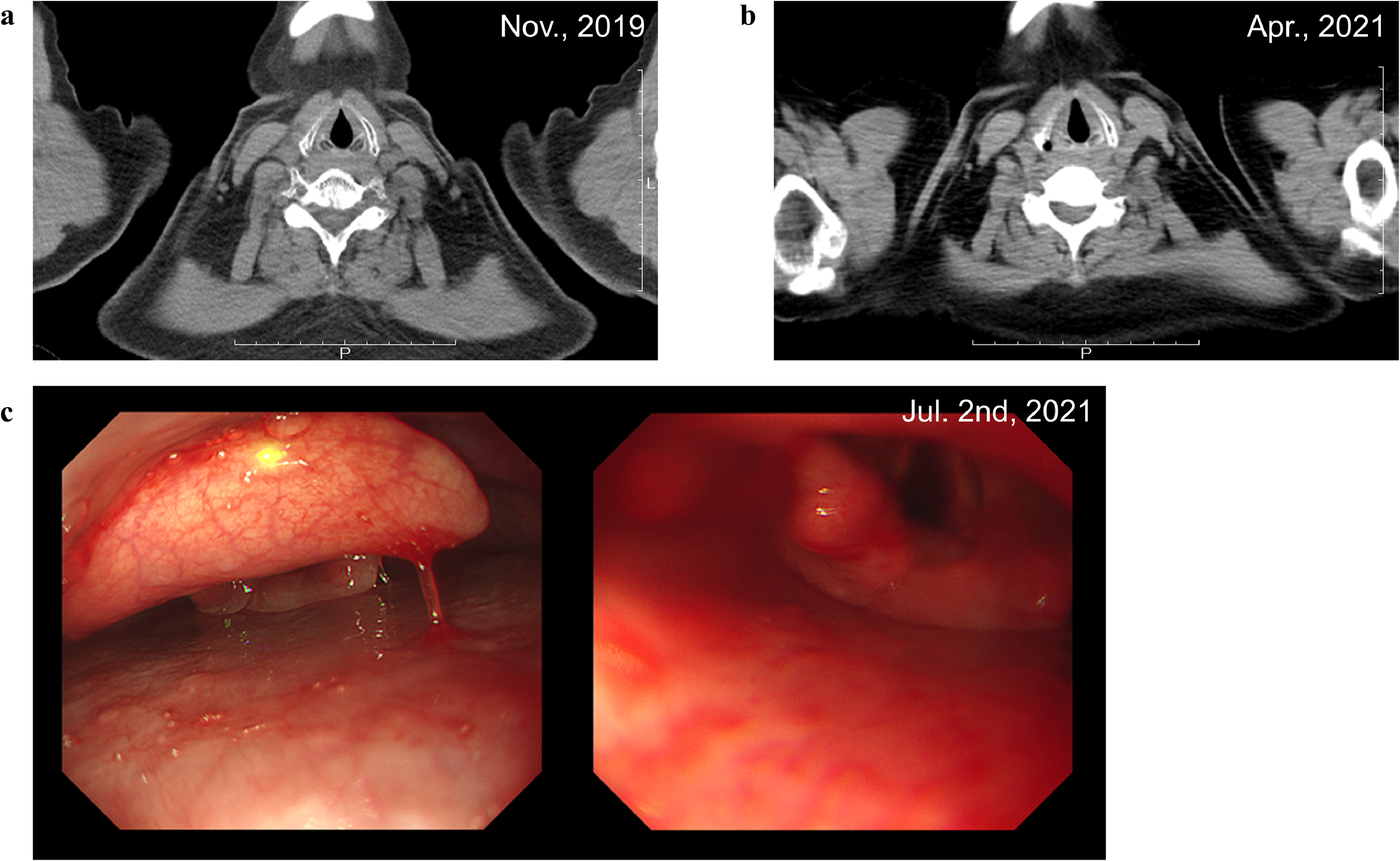

Supplement: Supplementary file 3 [file mmc3.jpg]
